# Infant formula and toddler milk marketing and caregiver's provision to young children

**DOI:** 10.1111/mcn.12962

**Published:** 2020-03-10

**Authors:** Maria J. Romo‐Palafox, Jennifer L. Pomeranz, Jennifer L. Harris

**Affiliations:** ^1^ Rudd Center for Food Policy and Obesity University of Connecticut Hartford Connecticut; ^2^ College of Global Public Health New York University New York New York; ^3^ Present address: Saint Louis University Saint Louis Missouri

**Keywords:** Expert recommendations, infant formula, marketing claims, policy, toddler milk

## Abstract

The World Health Organization International Code of Marketing of Breast‐milk Substitutes prohibits claims and other marketing that may confuse caregivers about benefits of formula and other milk‐based drinks for infants and toddlers, but such marketing is common in the United States. This study assessed caregivers' provision of milk‐based products to their infants and toddlers and potential confusion about product benefits and appropriate use. Online survey of 1,645 U.S. caregivers of infants (6–11 months) and toddlers (12–36 months). Respondents identified infant formula and toddler milk products they served their child (ren) and provided relative agreement with common marketing claims. Logistic regression assessed relationships between agreement and serving these products, controlling for individual characteristics. Over one‐half of caregivers of infants (52%) agreed that infant formula can be better for babies' digestion and brain development than breastmilk, and 62% agreed it can provide nutrition not present in breastmilk. Most caregivers of toddlers (60%) agreed that toddler milks provide nutrition toddlers do not get from other foods. Some caregivers of infants (11%) reported serving toddler milk to their child most often. Agreement with marketing claims increased the odds of serving infant formula and/or toddler milks. For caregivers of toddlers, odds were higher for college‐educated and lower for non‐Hispanic White caregivers. Common marketing messages promoting infant formula and toddler milks may mislead caregivers about benefits and appropriateness of serving to young children. These findings support calls for public health policies and increased regulation of infant formula and toddler milks.

**Key messages**

- Infant formula and toddler milk advertising may substantially affect caregivers' choice of milk for their child.
- Most caregivers believed marketing claims promoting unsubstantiated benefits of infant formula and toddler milks.
- Parents who believed these marketing claims were more likely to serve infant formulas or toddler milks to their child.
- Advertising that suggests infant formula and toddler milks provide developmental and other benefits for young children may neutralize public health messages and obscure health risks.
- Awareness of recommendations to offer toddlers whole milk and avoid sugary beverages did not prevent parents from providing toddler milks.
- Common infant formula and toddler milk marketing claims may mislead caregivers about product benefits and appropriateness for their child; public health campaigns to counter marketing claims and regulation of toddler milk labels are required.

## 1 INTRODUCTION

The World Health Organization (WHO) enacted the International Code of Marketing of Breast‐milk Substitutes (the Code) in 1981 to address inappropriate and potentially harmful marketing of breastmilk substitutes (BMS). The Code aims “to contribute to the provision of safe and adequate nutrition for infants, by the protection and promotion of breast‐feeding, and by ensuring the proper use of breast‐milk substitutes, when these are necessary, on the basis of adequate information and through appropriate marketing and distribution” (World Health Organization, 1981). Since 1981, the World Health Assembly has adopted subsequent resolutions to address Code implementation and new developments in BMS marketing (WHO, 2018). For example, in 2016 the World Health Assembly adopted Resolution 69.9 that expanded the definition of BMS to cover marketing of complementary foods, including liquid or powdered milk‐based products intended for children up to age 3 years (known by various names, including toddler milks, growing‐up milks, and toddler formula; World Health Organization, 2016). The Code addresses a variety of marketing practices that may discourage breastfeeding and calls for prohibiting all advertising and promotion of BMS to the general public and through the health care system (Marketing of Breast‐Milk Substitutes: National Implementation of the International Code Status Report 2018, 2018). The Code also prohibits messages that compare BMS with breastfeeding or idealize formula feeding; nutrition and health claims; and manufacturer‐sponsored breastfeeding advice.

However, the United States is one of just six countries that has not enacted any provisions of the Code either by legal or by voluntary measures (Marketing of Breast‐Milk Substitutes: National Implementation of the International Code Status Report 2018, 2018). Research has demonstrated widespread marketing of infant formula and toddler milks directly to U.S. consumers through practices prohibited under the Code. Common practices include advertising on TV and other media, nutrition and health claims, and messages that imply that benefits of infant formula over breastfeeding (Basch, Shaffer, Hammond, & Rajan, 2013; Harris et al., 2016; Huang, Labiner‐Wolfe, Huang, Choiniere, & C.,, & Fein, S. B., 2013; Mejia, Selkir, Gardin, & Nixon, 2016; Stang, Hoss, & Story, 2010). Furthermore, infant formula and toddler milk marketing features numerous structure/function claims that imply that product ingredients (e.g., Docosahexaenoic Acid (DHA), reduced lactose, and pre/probiotics) will benefit young children's brain and cognitive development, digestive health (i.e., reduce fussiness, gas, or colic), and/or immunity (Abrams, 2015; Belamarich, Bochner, & Racine, 2016; Pomeranz, Romo Palafox, & Harris, 2018). Scientific evidence does not support the accuracy of structure/function claims commonly found on these products, such as the link between provision of DHA through infant formula and brain development or between prebiotics and the immune system (Belamarich et al., 2016; Food and Drug Administration, 2016; Hughes, Landa, & Sharfstein, 2017). Although the U.S. Food and Drug Administration (FDA) does not require that such claims be supported by scientific evidence it has recommended that infant formula manufacturers substantiate structure/function claims to ensure they are truthful and not misleading (FDA, 2016; Hughes et al., 2017).

Experts have long raised concerns that infant formula marketing misleads caregivers to believe that it is equivalent to, or better than, breastmilk (Belamarich et al., 2016; Hughes et al., 2017; Marketing of Breast‐Milk Substitutes: National Implementation of the International Code Status Report 2018, 2018). More recently, experts also have expressed concerns about potential misleading marketing of toddler milks (Lott, Callahan, Welker Duffy, Story, & Daniels, 2019; Pomeranz et al., 2018). These products typically consist of fat‐free cow's milk, vegetable oils, and added caloric sweeteners (e.g., corn syrup, lactose, and sucrose). In a consensus statement on healthy beverages for young children, issued by leading U.S. health organizations in 2019, experts do not recommend serving toddler milks (Lott et al., 2019). They raised concerns about the added sugars in these products and concluded that toddler milks provide no unique nutritional value, also noting that toddler milk costs more than cow's milk, which is recommended for young children. Nonetheless, toddler milks are the fastest growing “formula” category in the United States (Harris et al., 2016).

Furthermore, qualitative research indicates considerable consumer confusion regarding the difference between infant formulas and toddler milks, which may lead to their inappropriate provision (N. Berry, Jones, & Iverson, 2010; Cattaneo et al., 2015). The FDA has not established a consistent term for toddler milks (i.e., standard of identity) or nutrition requirements, and manufacturers use differing terms on packaging, including “toddler formula,” “milk drink,” and “toddler drink,” as well as various formulations (Pomeranz et al., 2018; Pomeranz & Harris, 2019). Furthermore, cross‐promotion of infant formula and toddler milks from the same manufacturers (i.e., similar packaging and branding) raises concerns that caregivers may mistakenly provide toddler milks to infants (which do not meet infants' nutritional requirements) or believe that the “toddler” version of an infant formula is the appropriate “next” product for children over 12 months (Pereira et al., 2016; Pomeranz et al., 2018). Toddler milks are also less expensive and sometimes stocked near infant formula on product shelves, raising further concerns about inappropriate provision to infants (Harris et al., 2016). However, quantitative research has not examined potential confusion about toddler milks among U.S. caregivers.

This exploratory study aimed to quantitatively assess consumer confusion about the difference between infant formula and toddler milks and examine the potential impact of common marketing claims on caregivers' provision to their infants and toddlers (6–36 months). Specific study objectives include measuring caregivers' provision of infant formula and/or toddler milks to their children; documenting the proportion who provided a product not intended for their child's age (i.e., infant formula to toddlers or toddler milks to infants); and assessing caregivers' agreement with common marketing claims and expert recommendations about milk‐based products for young children. We hypothesized that agreement with common infant formula and toddler milk marketing claims would be associated with caregivers' provision of these products to their young children.

## 2 METHODS

We conducted a cross‐sectional online survey of U.S. parents and other primary caregivers of infants (6–11 months) and toddlers (12–36 months) to assess (a) the food and drink products caregivers served their child (including category, brand, and product); (b) caregivers' agreement with expert recommendations on how and what to feed their child; and (c) caregivers' agreement with marketing messages (including product claims) used to promote infant formula and toddler milks. We recruited a large nonprobability sample (*N* = 1,645) to obtain a diverse cross‐section of participants.

### 2.1 Survey participants

We utilized one nationally representative online survey panel (Innovate, 2016) and another panel of U.S. Hispanic households (Offerwise, 2016) to recruit participants who have enrolled in the panels and agree to participate in online surveys. Innovate recruits panel members through in‐app banner advertising, social networks such as Facebook, and numerous web and SMS databases (Innovate, 2016). Offerwise recruits panel members through TV advertising on both Spanish‐ and English‐speaking networks (Innovate, 2016; Offerwise, 2016). To ensure the quality of respondents, the panels do not provide monetary incentives for responding to individual surveys, but members receive points when they complete a survey. The number of points varies based on the length of the survey and the difficulty of recruiting participants that meet survey requirements. These points accumulate and can be redeemed for a variety of incentives, such as online gift cards and charitable donations.

For our survey, the panel companies invited panel members with young children (age 3 years and under) to participate in the survey and sent them a link to the survey if they agreed (participation was voluntary). The survey screened for primary caregivers of at least one 6‐ to 36‐month‐old child who had decision‐making responsibility regarding what to feed their child. We established quotas to achieve equal numbers of respondents by child's age group (infants: 6–11 months; young toddlers: 12–23 months; older toddlers: 24–36 months), as well as by minimum numbers of Black and Asian caregivers (from the general panel) and higher and lower acculturation levels (Hispanic panel). Due to relatively low prevalence of some demographic groups (e.g., Asian parents with children 6–36 months) within the U.S. adult population, quota sampling was used to recruit a sufficient sample to allow for meaningful comparisons between all groups of interest (Bornstein, Jager, & Putnick, 2013). Data collection occurred from April to June 2017.

### 2.2 Survey design and pretest

Participants answered the 30‐min online survey delivered via Qualtrics survey software (Qualtrics, 2005). We pretested survey items using a convenience sample of 20 caregivers of infants and toddlers (6–36 months old). As outlined by Czaja and colleagues, pretest objectives and techniques include one‐on‐one cognitive interviews where participants think out loud while responding to each survey question and then answer probes to identify comprehension, interpretation, and recall problems (Czaja & Blair, 2005; Presser et al., 2004). The Institutional Review Board of the [deidentified University] approved all measures and procedures.

### 2.3 Measures (see Data S1 for specific survey items)

#### 2.3.1 Selection of child

Participants reported the age and gender of child (ren) living in their household between 6 and 36 months of age. Children with a disease or condition that requires a special diet (e.g., lactose intolerance) were excluded (*n* = 233). Participants with more than one eligible child selected the child with the most recent birthday and answered questions about that child. We divided participants into three groups according to the age of the child they selected: infants (6–11 months), young toddlers (12–23 months), and older toddlers (24–36 months).

#### 2.3.2 Milk‐based product categories and specific products served

Due to potential confusion about the meaning of product category names (i.e., “infant formula” vs. “toddler milks”) and similarity of brand names for different products (e.g., Enfamil infant formula vs. Enfagrow toddler milk), first we assessed the categories of milk‐based products that participants served their child. We then used a two‐step process to identify the specific infant formula or toddler milk product they served most often.

Participants first selected *all categories* of milk drinks they had served their child in the past month, from four options: “Infant formulas (e.g., Enfamil, Gerber Good Start, Similac),” “Other formulas or powdered milks (e.g., Enfagrow, Gerber Good Start Grow, Nido 1+, Similac Go & Grow),” “Regular milk (i.e., cow's),” and “Non‐dairy milk (e.g., almond, coconut, soy).” The survey provided examples of infant formula and toddler milk brand names for clarity. Due to the lack of a commonly used term for “toddler milks,” the survey described them as “Other formulas or powdered milks.” The survey also included options for “Other” (with a fill‐in box) and “None of the above” (including if they only served breastmilk).

To identify the *product served most often*, if participants selected “Infant formulas” and/or “Other formulas or powdered milks” in the category question, the next question showed a list of infant formula and toddler milk brand names and logos. Participants then selected all brands they had served their child in the past month. The list of brand names and logos included all infant formula and toddler milk brands that had spent $100,000 or more in total advertising in 2015, as identified in previous research (Harris et al., 2016). The question also included an “Other” option for participants to write in a different brand.

Based on the brands selected in the previous question, participants then saw a list of all specific products (with package images and names) offered by each of the brands selected, and they chose the product they had served most often in the past month to their child. The list included all individual products offered by the brands in the previous question (including all varieties of both infant formula and toddler milks), but excluded products for specific dietary needs (e.g., pre‐term infants and protein allergies; Harris et al., 2016). The survey grouped brands of infant formula and toddler milks with similar names (e.g., Enfamil and Enfagrow and Happy Baby and Happy Tot) as one brand (see Data S1 for specific brands and products included).

We created dichotomous variables to indicate whether participants had provided each of the following categories of milk to their child in the past month: infant formula, other formulas or powdered milk, regular milk, non‐dairy milk, and “none of the above” (which also included participants who had only provided breastmilk). We also identified participants who selected a product that was not intended for their child's age (i.e., infant formula for toddlers or toddler milk for infants) as the one provided most often to their child.

#### 2.3.3 Agreement with expert recommendations and marketing claims

Participants also indicated their agreement with various statements and claims about infant formulas, toddler milks, and other drinks for either infants or toddlers (according to their child's age) using 7‐point Likert scales from *strongly disagree* to *strongly agree.* Specific recommendations and claims presented in the survey are included in the results.

The survey included two statements each regarding expert recommendations for breastfeeding (shown to caregivers of infants [6–11 months]) or drinks for toddlers (shown to caregivers of toddlers [12–36 months]). Survey participants with an infant provided their agreement with four marketing claims commonly found on regular infant formula packages. Toddler caregivers saw one toddler milk claim (adapted from common claims on toddler milks) and a factual statement that toddler milks often contain added sweeteners (Harris et al., 2016; Lott et al., 2019; Pomeranz et al., 2018; Pomeranz & Harris, 2019). We categorized responses to agreement with expert recommendations and marketing claims as *disagree* (1–3), *neutral* (4), or *agree* (5–7). Due to high covariance between the four infant formula marketing claims (α = 0.81), we averaged them to create one scale for agreement with infant formula claims.

#### 2.3.4 Parent demographics

Participants reported their own gender, age, marital status, highest level of education, race, ethnicity, and household income. We categorized participants into the following racial/ethnic groups: non‐Hispanic White, non‐Hispanic Black, Hispanic, Asian, and mixed/other. Additionally, participants who selected Hispanic ethnicity answered the Short Acculturation Scale for Hispanics (SASH). This validated tool provides a score that assesses language preference, ranging from 1 (only Spanish) to 5 (only English). In accord with SASH methodology, we classified Hispanic participants as less acculturated (score <3.0) or more acculturated (Hamilton et al., 2009; Marin, Sabogal, Marin, Otero‐Sabogal, & Perez‐Stable, 1987). We included acculturation in the analysis due to cultural differences in young child feeding behaviours (Bigman, Wilkinson, Pérez, & Homedes, 2018; Cartagena et al., 2015; Dancel et al., 2015; Harris et al., 2016). In addition, two toddler milk brands advertised extensively on Spanish‐language TV (Harris et al., 2016).

### 2.4 Statistical analysis

We used Statistical Analysis System software for analyses (SAS System (Version 9.4), 2013). In addition to reporting descriptive statistics, including demographics, milk categories served to their child, and agreement with expert recommendations and marketing claims, we estimated a logistic regression model to predict whether caregivers served infant formula and/or toddler milk to their child in the past month. For this model, we combined caregivers who reported serving infant formulas and/or other formulas or powdered milks into one binary dependent variable to account for potential confusion between categories. Independent variables in the model included agreement with each expert recommendation (1–7), the scale to assess agreement with the infant formula marketing claims or agreement with the toddler milk marketing claim (1–7; depending on the child's age), caregiver education and race/ethnicity, age of child (months), and serving other milk types. As some caregivers served multiple types of milk to their infant or toddler, we wanted to assess the relationships between serving different types of milks and serving infant formula and/or toddler milk. We estimated separate models for caregivers of infants and toddlers.

## 3 RESULTS

Of 2,581 original participants, 23% did not meet inclusion criteria. The final sample totalled 1,645, for an 82% completion rate. The sample was mostly female (80%), with approximately equal numbers by child age group (i.e., infants, young toddlers, and older toddlers; Table 1). Due to quota sampling procedures used to obtain minimum numbers of Black and Hispanic participants, the sample was highly diverse (33% non‐Hispanic White).

**Table 1:** Sample characteristics

	Age of child						Total	
	Infants (6–11 months)		Young toddlers (12–23 months)		Older toddlers (24–36 months)			
	n	(%)	n	(%)	n	(%)	n	(%)
**Total sample**	555	100	556	100%	534	100	1,645	100
**Caregiver race/ethnicity**								
Non‐Hispanic White	157	28	190	34	190	36	537	33
Non‐Hispanic Black	171	31	120	22	76	14	367	22
Hispanic: more acculturated	69	12	83	15	87	16	239	15
Hispanic: less acculturated	82	15	72	13	114	21	268	16
Asian	65	12	80	14	51	10	196	12
Mixed/other	11	2	11	2	16	3	38	2
**Caregiver gender**								
Female^a^	441	79	438	79	430	81	1,309	80
Male	110	20	115	21	97	18	322	20
**Household income** a								
Under $40,000	245	44	252	45	252	47	749	46
$40,000 ‐ $74,000	174	31	159	29	160	30	493	30
$75,000 and over	131	24	143	26	121	23	395	24
**Caregiver education**								
High school or GED	94	17	116	21	128	24	338	21
Some college or 2‐year college	210	38	187	34	188	35	585	36
College graduate or higher	251	45	253	46	218	41	722	44
**Milk categories served to child in the past month** b								
Infant formula	406	75	315	58	227	44	948	59
Other formulas or powdered milk	122	22	239	44	213	41	574	36
Regular milk	109	20	347	64	327	63	783	49
Non‐dairy milk	39	7	101	19	103	20	243	15
None of the above	111	20	11	2	16	3	138	9

a Does not total to 100% due to missing data.

b Totals more than 100% due to multiple responses.

Although most participants reported serving their child the type of milk appropriate for their age (i.e., infant formula for infants and regular milk for toddlers), 22% of caregivers of infants (6–11 months) indicated that they had provided “other formulas” (i.e., not infant formula) in the past month. In addition, 50% of caregivers of toddlers indicated they had served their child infant formula. A smaller number of toddler caregivers (41%) indicated that they had served other formulas (i.e., the toddler milks) in the past month. Thirty‐nine percent of all toddler caregivers served regular and/or non‐dairy milks in addition to infant formula and/or toddler milks products; whereas 29% served only regular and/or non‐dairy milk only.

### 3.1 Potential confusion

When asked to select the specific product they served most often in the past month, not all caregivers indicated a product that was appropriate for their child's age. Among all caregivers of infants surveyed, 11% chose a toddler milk as the product they served most often. However, 53% had only selected “infant formula” when asked what categories of milk products they served, indicating that they may have believed the toddler milk product they served most often was an infant formula. Among toddler caregivers surveyed, 39% chose an infant formula as the product they served most often, but only 78% of these participants correctly categorized the product they had served as an infant formula.

### 3.2 Agreement with expert recommendations and marketing claims

The majority of caregivers agreed with all expert recommendations and marketing claims (Table 2). Although more than 80% of infant caregivers surveyed agreed with expert recommendations about the superiority of breastfeeding, 62% of infant caregivers also agreed that “infant formulas can provide nutrition that babies do not get from breastmilk” and 52% of infant caregivers agreed that “infant formulas can be better for babies' digestion and brain development than breastmilk.” Most toddler caregivers (73%) also agreed with experts that toddlers should drink plain whole milk and not drink products with added sugar. Yet 60% also agreed that toddler formulas provide nutrition that toddlers do not get from other food and drinks, despite broad agreement (72%) that toddler formulas or powdered milk often contain added sweeteners. The combined scale demonstrated strong agreement with infant formula marketing claims (*M* = 4.6, *SD* = 1.4).

### 3.3 Associations with serving infant formula and/or toddler milk

**Table 2:** Caregivers' agreement with expert recommendations and marketing claims

Infants: 6–11 months (*n* = 544)			
Expert recommendations	Disagree (%)*	Neutral (%)*	Agree (%)*
It is best to breastfeed infants until they are at least 12 months old.	7	12	81
Breastmilk provides all the nutrition a child under 6 months needs.	4	6	90
Marketing claims			
Infant formulas can provide nutrition that babies do not get from breastmilk.	27	12	62
Infant formulas can be better for babies' digestion than breastmilk.	31	17	52
Infant formulas can be better for babies' brain development than breastmilk.	33	16	52
Infant formulas help babies grow	5	11	84

All toddlers: 12–36 months (*n* = 1,066)			
Expert recommendations and product information	Disagree (%)*	Neutral (%)*	Agree (%)*
Children under age 2 years should not consume any drinks with added sugars.	10	15	76
Children between 1 and 2 years old should drink plain whole milk.	12	15	73
Toddler formulas or powdered milks often contain added sweeteners.	11	17	72
Marketing claim			
Toddler formulas or powdered milks provide nutrition that toddlers do not get from other food and drinks.	9	31	60

* Likert scale from Disagree (1–3) Neutral (4) to Agree (5–7).

Table 3 presents results of the logistic regression models to examine associations between provision of infant formula/toddler milk and participants' agreement with expert recommendations and marketing claims, controlling for demographic variables and other types of milks served.

**Table 3:** Odds that caregiver served infant formula and/or toddler milk in the past month via agreement with statements and demographic characteristics

Infants (6–11 months; *n* = 544)	Odds ratio	Confidence interval
Agreement with expert recommendations^a^		
It is best to breastfeed infants until they are at least 12 months old.	0.95	(0.78, 1.16)
Breastmilk provides all the nutrition a child under six months needs.	0.95	(0.80, 1.12)
Agreement with marketing claims^a^^,^ d	1.45^***^	(1.23, 1.70)
Education		
High school or GED (reference group)	—	—
Some college or 2‐year college	1.09	(0.58, 2.03)
College graduate or higher	0.66	(0.36, 1.22)
Race/ethnicity		
White non‐Hispanic (reference group)	—	—
Black non‐Hispanic	1.99	(1.09, 3.64)
Hispanic: more acculturated	1.34	(0.70, 2.54)
Hispanic: less acculturated	1.18	(0.61, 2.27)
Asian	2.27	(0.90, 5.76)
Age of child in months^b^	1.01	(0.88, 1.16)
Served other milk categories^c^		
Regular milk	4.10**	(1.66, 10.12)
Non‐dairy milk	8.52*	(1.11, 65.26)

All toddlers (12–36 months; *n* = 1,066)	Odds ratio	Confidence interval
Agreement with expert recommendations and product statement^a^		
Children under age two should not consume any drinks with added sugars.	0.83^**^	(0.74, 0.93)
Children between 1 and 2 years old should drink plain whole milk.	0.98	(0.88, 1.10)
Toddler formulas or powdered milks often contain added sweeteners.	1.09	(0.96, 1.24)
Agreement with marketing claim^a^		
Toddler formulas or powdered milks provide nutrition that toddlers do not get from other food and drinks.	1.59^***^	(1.41, 1.79)
Education		
High school or GED (reference group)	—	—
Some college or 2‐year college	1.08	(0.73, 1.61)
College graduate or higher	1.64^*^	(1.09, 2.46)
Race/ethnicity		
White non‐Hispanic (reference group)	—	—
Black non‐Hispanic	2.87^***^	(1.84, 4.47)
Hispanic: more acculturated	1.79^***^	(1.13, 2.84)
Hispanic: less acculturated	1.52	(0.99, 2.34)
Asian	4.09^***^	(2.36, 7.09)
Age of child in months^b^	0.95^***^	(0.94, 0.97)
Served other milk categories^c^		
Regular milk	0.18^***^	(0.12, 0.26)
Non‐dairy milk	1.41	(0.96, 2.07)

*Note.* Seventy‐four percent of infants and 66% of toddlers selected an infant formula or toddler milk product.

a Change in odds for each additional point of agreement (1–7 scale).

b Change in odds for each additional month of child's age.

c Reference group is participants who did not serve the milk category to their child.

d Average agreement with four regular infant formula claims, α = 0.81.

* *p* < .05 level.

** *p* < .01 level.

*** *p* < .001 level.

In the model for caregivers of infants, when holding all other variables constant, the odds of serving infant formula/toddler milk increased by 45% with each point on the scale of agreement with infant formula marketing claims (from 1 [*strongly disagree*] to 7 [*strongly agree*]). The odds of serving infant formula/toddler milk were four times higher if caregivers also served regular milk to their infant and 8.5 times higher if they also served non‐dairy milk. Neither agreement with expert recommendations, parent education, or race/ethnicity, nor infant age in months were associated with serving infant formula/toddler milk.

The model for caregivers of toddlers also found a strong association between agreement with marketing claims and serving infant formula/toddler milk. When holding all other variables constant, the odds of serving them increased by 59% with each point of agreement with the toddler milk marketing claim. The odds of serving infant formula/toddler milk also decreased by 17% with each point of agreement that “children under age two should not consume drinks with added sugars.”

In contrast to the infant caregiver model, we found some demographic differences in the odds of toddler caregivers serving infant formula/toddler milk. College graduates were 62% more likely to serve these products than caregivers with a high school degree or less. Caregiver race/ethnicity also predicted serving infant formula/toddler milks.

Asian, non‐Hispanic Black, and more‐acculturated Hispanic (but not less acculturated) participants were more likely to serve these products compared with non‐Hispanic White caregivers. Odds also decreased by 5% for every additional month of their child's age. In addition, odds were significantly lower if caregivers served their child regular milk (OR = 0.18). However, neither agreement that children should drink plain whole milk nor understanding that toddler milks contain added sweeteners was significantly related to caregivers' provision of infant formula/toddler milks to toddlers.

## 4 DISCUSSION

To our knowledge, this study is the first to demonstrate a relationship between agreement with infant formula and toddler milk marketing claims and whether caregivers served these products to their infant or toddler. Although the majority of participants agreed with expert recommendations to serve breastmilk to infants and plain whole milk to toddlers, it appears that they do not believe that those recommendations contradict common marketing claims about the benefits of infant formula over breastmilk and toddler milks over other food and drinks, as the majority of participants also agreed with marketing claims. Demographic characteristics were not related to serving infant formula/toddler milk to infants, but more educated caregivers, as well as non‐Hispanic Black, Asian, and more‐acculturated Hispanic caregivers, were more likely to serve them to their toddlers.

These results also provide additional evidence that caregivers may be inappropriately serving toddler milks to infants. In this sample, 11% of infant caregivers reported serving a toddler milk product most often to their infant. This practice raises substantial concerns as toddler milks do not contain the required nutrition for infants (Lott et al., 2019). It also supports concerns that cross‐branding of infant formula and toddler milks may confuse caregivers (N. Berry et al., 2010; Cattaneo et al., 2015; Pereira et al., 2016), as more than one‐half of those offering toddler formula to infants appeared to believe they were serving an infant formula.

Furthermore, this study demonstrates widespread provision of infant formulas to toddlers, as has been reported in previous research (Berry et al., 2010). Although it appears that the majority of toddler caregivers who served infant formula understood that it was an infant formula, this practice is contrary to recommendations to serve plain regular milk at this age (Lott et al., 2019).

In addition to their increased likelihood to serve infant formula/toddler milks to their child, toddler caregivers with a college degree in this sample were more likely to agree with the toddler milk marketing claim. This finding indicates that misperceptions about the meaning of marketing claims are not due to lower levels of education or literacy. In addition, the finding that non‐Hispanic Black, more‐acculturated Hispanic, and Asian caregivers were more likely to report serving infant formula/toddler milk to toddlers supports research in other domains suggesting that minority consumers may be more influenced by brand marketing than non‐Hispanic White consumers (Baby Food and Drink—US ‐ March 2018 *Nutrition Leads Drink Concerns*, 2018). However, it is not clear why less‐acculturated Hispanic caregivers were not more likely to serve these products.

It is also notable that caregivers who served regular and/or non‐dairy milk to infants were more likely to also serve infant formula/toddler milk, suggesting potential confusion about the adequacy of providing only infant formula or breastmilk (as recommended) for children up to 12 months. In contrast, toddler caregivers who served regular milk were less likely to serve infant formula/toddler milk, which indicates that caregivers may perceive toddler milk products to be appropriate substitutes for regular milk.

### 4.1 Opportunities for health professional guidance

Although most caregivers in this survey agreed with expert recommendations about the superiority of breastfeeding over infant formula and the need to introduce young children to plain milk at approximately 1 year and avoid products with added sugar, they also agreed with common marketing claims that compare infant formula favourably to breastmilk and promise nutritional benefits from serving toddler milk. This finding suggests a need for health care providers and the public health community to specifically refute these marketing claims and clearly communicate that infant formula and toddler milk have no advantages over breastmilk and regular milk. The 2019 consensus statement by leading U.S. health organizations, including the American Academy of Pediatrics and the Academy of Nutrition Dietetics, specifically recommended against serving toddler milks, which represents an important first step. Similar guidance about infant formulas that promote unproven claims about benefits for common infant conditions, such as gas and fussiness, also appear to be necessary. Furthermore, public health campaigns and health care provider training should address and attempt to correct caregivers' misunderstandings about potentially misleading claims used in infant formula and toddler milk marketing.

### 4.2 Opportunities for public health policy

These findings also demonstrate the need for more effective regulation of infant formula and toddler milk marketing in the United States. The FDA has the authority to regulate product labelling and could take action to address potentially misleading marketing claims and confusion about the difference between infant formula and toddler milk (Pomeranz & Harris, 2019). For example, the FDA issued draft guidance that structure/function claims on infant formula should be substantiated by scientific evidence. It should enact this guidance, establish the same requirements for toddler milk, and not allow claims that compare infant formula with breastmilk (which was specifically omitted from the FDA draft guidance). Such comparisons run contrary to WHO recommendations. Half of the respondents in our sample believed that infant formula is actually *better* than breastmilk, suggesting that these claims are misleading or deceptive.

The FDA should also enact new regulations for toddler milk labelling requirements (Pomeranz & Harris, 2019). One reason that caregivers may inappropriately serve toddler milk to infants and infant formula to toddlers could be due to confusion about the appropriate ages to serve each product and/or difficulty in distinguishing between the two categories, as identified in this research and previous qualitative studies (N. Berry et al., 2010; N. J. Berry, Jones, & Iverson, 2012; Rigo, Willcox, Spence, & Worsley, 2018). The FDA should establish a common name for toddler milks (i.e., statement of identity) and require that toddler milk labels clearly state the appropriate ages for the product and that they are not substitutes for infant formula (Pomeranz et al., 2018). Nutrient requirements may also be necessary for toddler milks (as currently required for infant formula) given the high rate of use by caregivers of both infants and toddlers.

### 4.3 Limitations and strengths

This study was cross‐sectional, so it does not provide evidence that marketing claims or confusion about product categories caused caregivers to serve infant formulas or toddler milks to their children. Although we took great care to avoid using potentially unfamiliar terms (e.g., toddler milk) and ascertain the exact products that participants served their child, and pretesting indicated participant understanding of category names used (i.e., infant formula, other formula, and powdered milk), this online survey did not allow for additional probes to assess participants' understandings. In addition, as with all self‐reported data, responses are subject to potential recall and/or desirability biases.

Strengths of this study include the use of online survey panels, which offer geographic heterogeneity and the ability to assess differences by participant race/ethnicity, education, and income through sampling quotas. In addition, inclusion of actual brand and product names and images likely reduced misreporting of products served. Additional research is required to demonstrate that marketing claims caused caregivers to serve infant formula or toddler milk to their young child and that cross‐branding caused confusion about the appropriate products for their child's age. However, this study suggests that marketing of infant formula and toddler milk may substantially affect caregivers' decisions about the milk‐based products they serve their children.

### 4.4 Public health implications

Despite awareness of expert recommendations and public health efforts to promote breastfeeding for infants and plain whole milk for toddlers, the majority of caregivers agreed with common marketing claims that serving infant formula or toddler milks benefits their child. In addition, these messages may mislead caregivers to inappropriately provide them to young children. These findings support calls for increased regulation of marketing for infant formula and toddler milks. Public health organizations should provide additional guidance to caregivers about appropriate milk‐based products for infants and toddlers and highlight issues with potentially misleading marketing claims that imply these products are nutritionally superior and provide benefits over recommended milk‐based products for young children.

## CONFLICTS OF INTEREST

The authors declare that hey have no conflicts of interest.

## CONTRIBUTIONS

MJR‐P and JLH were responsible for the conception and design of the study, and acquisition of data. JLP also participated in the analysis and interpretation of data, drafting, and revising the manuscript. All authors have approved the version for publication.

## Supporting information

Data S1 Supporting Information
