# Supplementary material for: Infant formula and toddler milk marketing and caregiver's provision to young children
Source: Matern Child Nutr. 2020 Mar 10;16(3):e12962. doi: 10.1111/mcn.12962 (PMC7296786; doi:10.1111/mcn.12962)
Supplement: Supplementary file 1 — Data S1 Supporting Information [file MCN-16-e12962-s001.docx]

Parent Perceptions on Feeding Their Young Children 3-22-2017

Which of the following formulas or milk drinks have you served to your child? Please select all that you have served to your child **in the past month.**

- Infant formulas (such as Enfamil, Gerber Good Start, Similac)
- Other formulas or powdered milks (such as Enfagrow, Gerber Good Start Grow, Nido 1+, Similac Go & Grow)
- Regular milk (cow's)
- Non-dairy milk (almond, soy, coconut)
- Other (please specify)

- ⊗ None of the above

From the list below, please select all brands that you served **in the past month**.

-
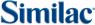

-
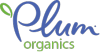

-
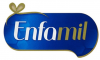

-
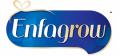

-
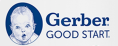

-
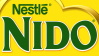

-
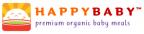

-
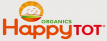

- Other (please specify)

- ⊗ **None of the above**

From the list below, please select the formula or powdered milk product that you served most often **in the past month.**

|  | Similac Advance Non-GMO 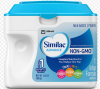 |  | Enfagrow Toddler Transitions 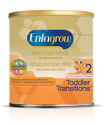 |
| --- | --- | --- | --- |
|  | Similac Advance 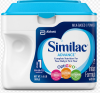 |  | Enfagrow Toddler Transitions Gentlease 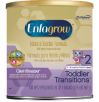 |
|  | Similac for Supplementation 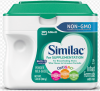 |  | Enfagrow Toddler Transitions Soy 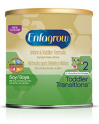 |
|  | Similac Organic 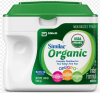 |  | Other Enfagrow product (please specify) |
|  | Similac Go & Grow NON-GMO 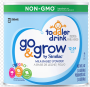 |  | Gerber Good Start Gentle for Supplementing 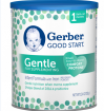 |
|  | Similac Go & Grow Sensitive 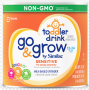 |  | Gerber Good Start Gentle 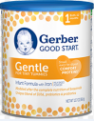 |
|  | Similac Go & Grow Vanilla 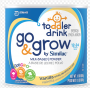 |  | Gerber Good Start Gentle 2 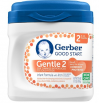 |
|  | Similac Go and Grow Milk 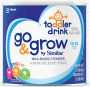 |  | Gerber Good Start Soothe 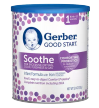 |
|  | Other Similac product (please specify) |  | Gerber Good Start Soy 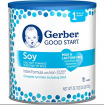 |
|  | Plum Organics Grow Well Organic 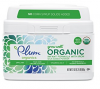 |  | Gerber Good Start 3 Soy 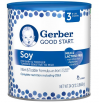 |
|  | Enfamil Enspire 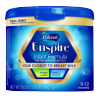 |  | Gerber Good Start Grow 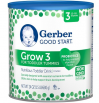 |
|  | Enfamil Newborn 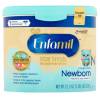 |  | Other Gerber product (please specify) |
|  | Enfamil Infant 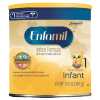 |  | Nestlé Nido Fortificada (Fortified) 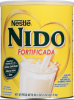 |
|  | Enfamil for Supplementing 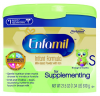 |  | Nestlé Nido Kinder 1+ 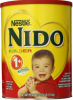 |
|  | Enfamil Gentlease 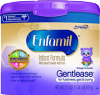 |  | Other Nestlé Nido product (please specify) |
|  | Enfamil Reguline 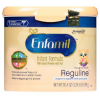 |  | Happy Tot Grow & Shine Organic Toddler Milk 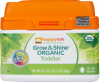 |
|  | Enfamil A.R. 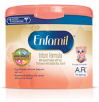 |  | Happy Baby Stage 1 Organic Infant Formula 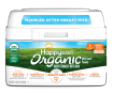 |
|  | Enfamil ProSobee 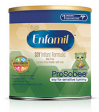 |  | Happy Baby Stage 2 Organic Infant Formula 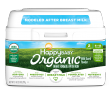 |
|  | Other Enfamil product (please specify) |  | ${q://QID26/ChoiceTextEntryValue/8} |
|  | Enfagrow Toddler Next Step 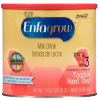 |  |  |

**FOR PARENTS OF INFANTS ONLY**

Please indicate how much you agree with the following statements about infant formula and breastfeeding.

|  | Strongly disagree | Disagree | Somewhat disagree | Neither agree nor disagree | Somewhat agree | Agree | Strongly agree |
| --- | --- | --- | --- | --- | --- | --- | --- |
| Infant formulas can provide nutrition that babies do not get from breastmilk. |  |  |  |  |  |  |  |
| Breastmilk provides all the nutrition a child under six months needs. |  |  |  |  |  |  |  |
| It is best to breastfeed infants until they are at least 12 months old. |  |  |  |  |  |  |  |
| Most infants do not need specialty formulas (e.g., sensitive, soy, soothe formulas). |  |  |  |  |  |  |  |
| Specialty formulas (e.g. Sensitive, Soy, For Spit-Up, Soothe) are not worth the additional expense for most babies. |  |  |  |  |  |  |  |
| Infant formulas can be better for babies’ digestion than breastmilk. |  |  |  |  |  |  |  |
| Infant formulas can be better for babies’ brain development than breastmilk. |  |  |  |  |  |  |  |
| Infant formulas help babies grow |  |  |  |  |  |  |  |

**FOR PARENTS OF TODDLERS ONLY**

Please indicate how much you agree with the following statements about **drinks for toddlers (12 - 36 months old):**

|  | Strongly disagree | Disagree | Somewhat disagree | Neither agree nor disagree | Somewhat agree | Agree | Strongly agree |
| --- | --- | --- | --- | --- | --- | --- | --- |
| Children under age two should not consume any drinks with added sugars. |  |  |  |  |  |  |  |
| Children between 1 and 2 years old should drink plain whole milk. |  |  |  |  |  |  |  |
| Toddler formulas or powdered milks provide nutrition that toddlers do not get from other food and drinks. |  |  |  |  |  |  |  |
| Toddler formulas or powdered milks often contain added sweeteners. (Enfagrow Toddler Next Step, Gerber Good Start Grow, Nido 1+, Similac Go & Grow, Happy Tot Grow & Shine, etc) |  |  |  |  |  |  |  |

**FOR HISPANIC PARTICIPANTS ONLY**

Which is your preferred language in the following situations?

|  | Only English | English more than Spanish | English and Spanish equally | Spanish more than English | Only Spanish |
| --- | --- | --- | --- | --- | --- |
| In general, what language(s) do you read and speak? |  |  |  |  |  |
| What language do you usually speak at home? |  |  |  |  |  |
| In what language do you usually think? |  |  |  |  |  |
| What language do you usually speak with your friends? |  |  |  |  |  |
